# Assessing the Performance of Non-Equilibrium Thermodynamic Integration in Flavodoxin Redox Potential Estimation

**DOI:** 10.3390/molecules28166016

**Published:** 2023-08-11

**Authors:** Giuseppe Silvestri, Federica Arrigoni, Francesca Persico, Luca Bertini, Giuseppe Zampella, Luca De Gioia, Jacopo Vertemara

**Affiliations:** Department of Biotechnology and Biosciences BtBs, University of Milano-Bicocca, Piazza dell’Ateneo Nuovo 1, 20126 Milan, Italy

**Keywords:** MD simulations, thermodynamic integration, redox potential, flavoproteins

## Abstract

Flavodoxins are enzymes that contain the redox-active flavin mononucleotide (FMN) cofactor and play a crucial role in numerous biological processes, including energy conversion and electron transfer. Since the redox characteristics of flavodoxins are significantly impacted by the molecular environment of the FMN cofactor, the evaluation of the interplay between the redox properties of the flavin cofactor and its molecular surroundings in flavoproteins is a critical area of investigation for both fundamental research and technological advancements, as the electrochemical tuning of flavoproteins is necessary for optimal interaction with redox acceptor or donor molecules. In order to facilitate the rational design of biomolecular devices, it is imperative to have access to computational tools that can accurately predict the redox potential of both natural and artificial flavoproteins. In this study, we have investigated the feasibility of using non-equilibrium thermodynamic integration protocols to reliably predict the redox potential of flavodoxins. Using as a test set the wild-type flavodoxin from *Clostridium Beijerinckii* and eight experimentally characterized single-point mutants, we have computed their redox potential. Our results show that 75% (6 out of 8) of the calculated reaction free energies are within 1 kcal/mol of the experimental values, and none exceed an error of 2 kcal/mol, confirming that non-equilibrium thermodynamic integration is a trustworthy tool for the quantitative estimation of the redox potential of this biologically and technologically significant class of enzymes.

## 1. Introduction

**Figure 1:**
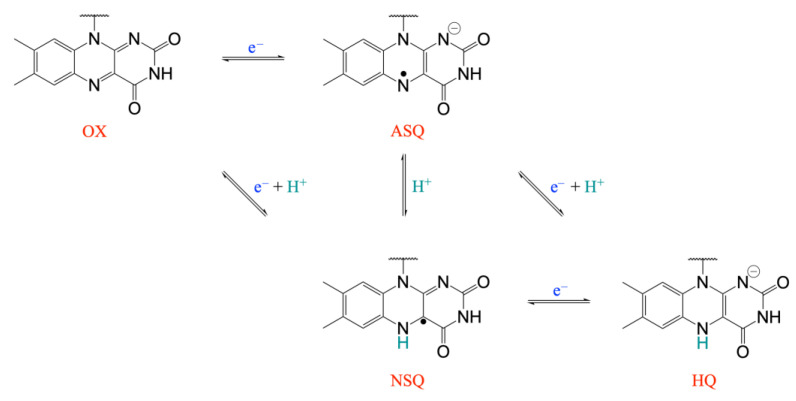
Redox and protonation states for the isoalloxazine ring in flavoproteins: quinone (OX), semiquinone (either as anionic, ASQ, or neutral, NSQ, species), and hydroquinone (HQ).

Due to the extraordinarily large number of known flavoproteins and the wide range of redox reactions that this family of enzymes may catalyze, flavoproteins rank among the most significant protein families engaged in redox processes [1,2]. According to the particular protein environment, the redox potential of flavoproteins can vary by hundreds of mV from the redox potential of the free flavin cofactor in water and span a very wide range [3,4]. The flavin cofactor is also peculiar because it can catalyze both one- and two-electron transfer reactions. In fact, flavins can exist in three redox forms (Figure 1): quinone (OX), semiquinone (either as anionic, ASQ, or neutral, NSQ, species), and hydroquinone (HQ). Since electrochemical tuning of flavoproteins is essential for optimal reactivity with acceptor or donor redox molecules, evaluating the relationships between the redox properties of the flavin cofactor and their molecular environment in flavoproteins is an important area of study for both basic research and technological applications [5,6,7,8]. In fact, the development of biosensors [9], biocatalysis [9,10], bioremediation [11], and bioelectronics [11] are just a few biotechnological applications that flavoproteins represent a potential molecular system for. Therefore, the availability of computational methods able to reliably predict the redox potential of natural and de novo designed flavoproteins is crucial for the rational design of tailored molecular devices.

Flavodoxins are a diverse group of flavoproteins that play a crucial role in a variety of biological processes, including electron transfer and energy conversion. These proteins contain a flavin mononucleotide (FMN) cofactor, which serves as a redox-active molecule and provides flavodoxin with its unique redox properties. In recent years, significant progress has been made in the study of flavodoxins and their redox properties. Structural and functional studies have established that electrostatic interactions are a dominant factor affecting the redox properties of the FMN cofactor. In particular, mutations in flavodoxins have revealed a strong correlation between the redox potential of the cofactor and the number of negatively charged groups in the vicinity of the flavin [12]. Structural and functional studies on flavodoxins have also established that in flavodoxin, the first reduction involves the transition from OX to NSQ, i.e., it can be described as a proton-coupled redox reaction [13,14,15,16], and electrostatic interactions are a dominant factor affecting the NSQ/HQ equilibrium. In particular, since the flavin hydroquinone in flavodoxins is not protonated at N1 [17], the isoalloxazine moiety is anionic, and it is expected to generate substantial repulsions in the negatively charged protein environment commonly observed in flavodoxins [18,19]. Indeed, mutations in *Desulfovibrio Vulgaris* flavodoxin have revealed a strong correlation of the NSQ/HQ potential with the number of negatively charged groups in the neighborhood of the flavin [19], confirming that the flavin mononucleotide cofactor bound to flavodoxins is more difficult to convert to the fully reduced form compared to free FMN. According to studies on wild-type and mutant flavodoxins from *Desulfovibrio vulgaris* and *Clostridium beijerinckii* [18,19,20,21], unfavorable aromatic stacking interactions can also be important in tuning the redox potential. Other investigations have also emphasized and shown the impact on the flavin reduction potential of particular hydrogen bonds, electrostatic, hydrophobic, and stacking interactions, as well as conformational alterations of the tricyclic ring or its surroundings [12,19,22,23,24,25]. However, it is still difficult to quantitatively evaluate the correlations between structural factors and redox properties, which limits the ability to rationally design synthetic flavoproteins with specific redox properties. Indeed, in order to estimate the redox potential of flavoproteins, a number of computational techniques have been evaluated and employed [26,27]. As a benchmark for subsequent QM/MM experiments aiming at examining the redox characteristics of tiny flavoproteins, Truhlar and associates presented a series of seminal DFT examinations of flavins in various solvents [28]. However, the systematic implementation of QM and QM/MM approaches is still hampered by the high computing cost of such calculations, despite the fact that QM and QM/MM studies enable predicting flavin reduction potentials with an average error of only 10–20 mV [29,30]. In addition to QM and QM/MM investigations, studies based on a description of flavoproteins in terms of molecular mechanics have also been published. A thermodynamic integration (TI) investigation was conducted by Sattelle and Sutcliffe [31] on a variety of naturally occurring and artificially created flavodoxins, each of which differed for one amino acid in the cofactor environment, obtaining an average difference between estimated and actual redox potential of 20–100 mV.

To further assess the possibility of using thermodynamic integration protocols to reliably predict the redox potential of flavodoxins, we have used a fast growth non-equilibrium approach to compute the redox potential of the flavodoxin from *Clostridium Beijerinckii*. In particular, we have conducted a systematic analysis of a number of single-point mutants of the flavodoxin from *Clostridium Beijerinckii*, for which redox potentials were already experimentally measured, with the aim of evaluating and validating this approach in the calculation of relative redox potential in flavoproteins, comparing results with similar investigations carried out using different methodological approaches. Non-equilibrium TI protocols have already been used to investigate other processes, such as docking of ligands to proteins and redox processes [32,33,34,35]. However, to the best of our knowledge, this is the first report about the evaluation of a non-equilibrium thermodynamic integration study for the prediction of the relative redox potential of biological macromolecules.

## 2. Results and Discussion

The accurate calculation of absolute protein redox potentials, i.e., within ~1 kcal/mol (~50 mV) of the experimental values, remains extremely challenging. As initially underlined by Van Gunsteren, Canters, and collaborators [36,37], and more recently by D’Abramo and collaborators [38], the direct computation of protein redox potential as a function of pH or protein composition using a comprehensive quantum-chemical treatment of the system is still essentially unattainable. In fact, such a task would necessitate a comprehensive quantum-chemical treatment of the reaction site, encompassing its immediate surroundings and accounting for entropic effects arising from alternative conformational states of the protein and the solvent. Consequently, researchers are compelled to adopt one or more simplifying assumptions to carry out such investigations. One viable approach involves employing high-level quantum-chemical methodology to characterize the redox center and its proximal environment while disregarding entropic contributions, the rest of the protein environment, and the solvent (or describing them using an MM forcefield). Alternatively, one may consider treating the protein in an explicit solvent using an empirical force field and classical dynamics. This approach incorporates entropic effects through extensive conformational sampling, treating oxidation and reduction as purely electrostatic phenomena and implicitly assuming that quantum-mechanical contributions are consistent across all mutations. A third possible approach involves the omission of both the quantum-mechanical nature of the transition and entropic contributions from the environment, treating oxidation and reduction solely as electrostatic phenomena. For example, conducting Poisson–Boltzmann calculations based on a given (X-ray) structure in the oxidized and reduced states.

With the aim of contributing to the evaluation of computational approaches for the calculation of protein thermodynamic properties, we have used the fast growth-TI method for the computation of flavodoxin’s redox potential, studying a dataset comprising the wild-type flavodoxin from *Clostridium beijerinkii* and eight single-amino acid mutants for which experimentally derived redox potentials are available.

The fast growth-TI method takes its roots from the Jarzynski equality [39,40], which relates the free energy difference (∆F) of states A and B to the work (*W*) done on the system to transform it from state A to state B:

$$e^{-\beta ∆F}=\langle e^{-\beta W}\rangle$$

where β is the reciprocal thermal energy.

The fast growth method relies on the Crooks equation [41], which relates the free energy difference with the forward (*W*) and reverse (*−W*) work done on a system during non-equilibrium transformation:

$$\frac{P_{f}(W)}{P_{r}(-W)}=e^{-\beta (W-∆F)}$$

Work values are calculated by integrating the Hamiltonians ($H_{A}$ and $H_{B}$) of the system with respect to a coupling parameter (λ) along the transition path:

$$W=\int_{0}^{1}\frac{\partial H}{\partial \lambda }\;\partial \lambda$$

The coupling parameter λ changes from 0 to 1 continuously and quickly to transform the system from state A to state B in order to obtain the forward work (*W*). The same happens to calculate the reverse work (*−W*), changing λ from 1 to 0. Both W and *−W* are calculated as an average over an ensemble of N trajectories that are started from an equilibrated canonical ensemble for both states A and B. In other words, a lot of very fast transitions (hundreds of transitions of a few tens of ns) are performed from A to B and vice versa, with a corresponding great saving of computational time.

It should be noted that, due to approximations to the energy function and possible incomplete sampling [42], the relative values of calculated molecular properties are generally more reliable than the corresponding absolute values. Therefore, with the aim of achieving better accuracy, the redox properties of single-point mutants have been evaluated using the wild-type value as a reference [31]. Specifically, ΔΔG values have been calculated by subtracting from the ΔG value of the WT the one relative to each mutant, according to the following formula:

ΔΔG_MUT_ = ΔG_MUT_ − ΔG_WT_

In addition, we have focused our attention on the OX/NSQ redox process, in which the reduction/protonation event is well characterized and directly involves the flavin cofactor (Figure 1). Of course, the computational investigation of the SQ/HQ redox process would also be relevant to the study. However, the protonation event associated with the SQ/HQ redox transition does not directly involve the flavin cofactor but has been suggested to imply the protonation of some aminoacids in the cofactor environment [23], making the protonation form of the protein to be used in the TI simulations more uncertain.

**Figure 2:**
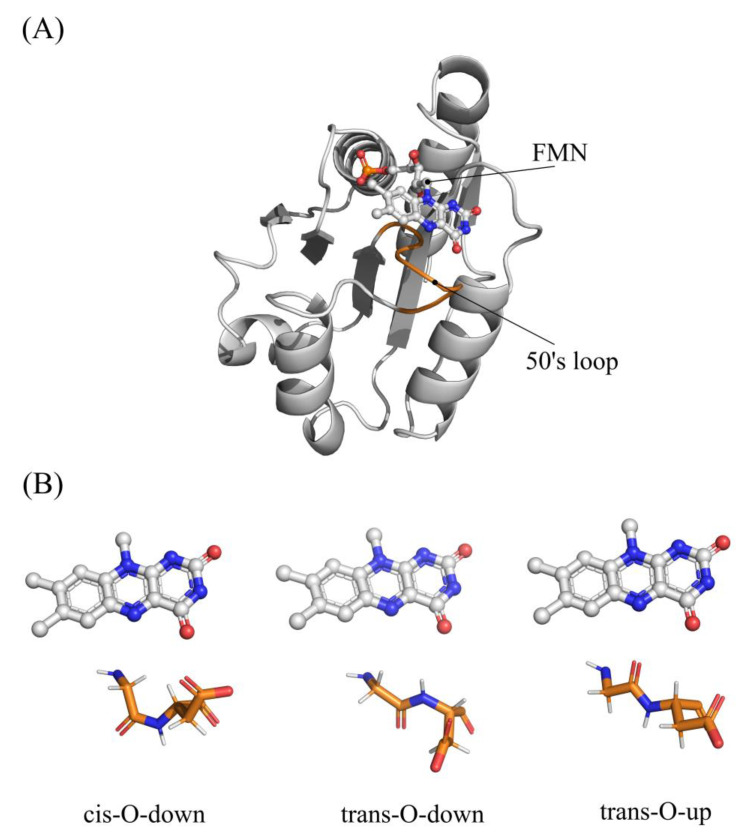
(**A**) structure of *C. Beijerinckii* flavodoxin (PDBID 5NLL). (**B**) Conformation of 50s loop.

All three redox states for the wild-type flavodoxin from *Clostridium beijerinckii* were previously characterized by X-ray diffraction [13]. It is noteworthy that the conformational properties of the so called “50s loop”, which includes the residues Met56-Gly57-Asp58-Glu59, have been suggested to be affected by the redox state of the isoalloxazine ring of the FMN cofactor [43]. In particular, the Gly57-Asp58 peptide bond in the OX redox state is found predominantly in the cis-O-down and trans-O-down conformations, which are characterized by the carbonyl group oriented opposite to the flavin ring (Figure 2).

After one-electron reduction, the peptide bond rearranges in a trans-O-up conformation where the carbonyl group, which plays the role of a hydrogen-bond acceptor, interacts with N5H, stabilizing the reduced FMN. In fact, according to X-ray data, the wild-type flavodoxin in the OX state is a mixture of cis-O-down (50%), trans-O-down (20%), and trans-O-up (30%) conformations. In light of these observations, we have initially evaluated the effect of the residues 57–58 conformation upon the OX/SQ reduction in the wild-type flavodoxin, taking into account these transitions:

FMN_OX/cis-O-down_ → FMN_NSQ/cis-O-down_

FMN_OX/cis-O-down_ → FMN_NSQ/trans-O-down_

FMN_OX/cis-O-down_ → FMN_NSQ/trans-O-up_

Three replicas of 10 ns were conducted on all four structures to obtain a representative ensemble for each state, then three replicas of the TI calculation were performed for each reaction, and the ΔG_OX→NSQ_ has been evaluated as the average of the replicas. The use of relatively brief MD replicas instead of longer MD trajectories as the source of conformational ensembles for the oxidized (OX) and reduced (SQ) states is based on previous findings by De Groot and colleagues, who reported that for obtaining reliable TI results, it is generally advantageous to sample conformations that are proximal to the experimentally determined structure [32]. Indeed, for the sake of completeness, we also tested the possibility of using 500 ns MD trajectories to generate starting points for the non-equilibrium TI simulations, but it turned out that the results were poorer (see below). The starting structures were retrieved from the pdb files 5NLL and 2FOX for OX-flavodoxin and SQ-flavodoxin, respectively. Results show very similar reaction free energy values in the three cases: ΔG_OX→NSQ_ of −42.92 kcal/mol, −42.91 kcal/mol, and −43.76 kcal/mol for the OX_(cis-O-down)_/NSQ_(trans-O-up)_, OX_(trans-O-down)_/NSQ_(trans-O-up)_, and OX_(trans-O-up)_/NSQ_(trans-O-up)_ redox transitions, respectively (Table 1). All values are in a range of 0.85 kcal/mol, suggesting that the OX/NSQ reaction energy is not strongly affected by the conformation of residues 56–57. 

In light of this observation, to simplify and reduce the number of simulations, we have used only the cis-O-down conformation as the OX state for all the flavodoxin variants for which the crystallographic structure is presently not available. The same applies to the NSQ state, for which we adopted the trans-O-up conformation.

Computed RMSD and RMSF values (Figures S1 and S2; Supplementary Materials) highlight the great structural stability of these proteins. In fact, the analysis of RMSF values allowed us to observe that the only protein portion undergoing conformational transitions along the MD simulations is the loop in front of FMN. This observation is relevant because the conformation of this loop is known to be important for the modulation of the flavin redox potential [43]. The calculated ΔΔG values for the OX to NSQ redox transition for the wild-type flavodoxin as well as for the single-point mutant investigated are collected in Table 2, and the corresponding distributions of forward and reverse work values (W) obtained during the TI simulations are shown in Figures S3 and S4; Supplementary Materials. Computed ΔΔG values obtained from 10n replicas can be generally considered satisfactory, with the minimum, maximum, and mean absolute error equal to 0.60, 3.00, and 1.37 kcal/mol, respectively. The method therefore fails at most by about 3 kcal/mol (G57T mutant), while the calculated RMSE is less than 0.93 kcal/mol. It is also very encouraging that the mean deviation of the calculated values from the experimental ones is only 31 mV. In fact, our results nicely compare to those obtained in a MD-FEP study carried out on *Anabaena*’s *Flavodoxins* [31] and also with the results reported by Grater and coworkers, who used a free-energy calculation scheme based on the Crooks Gaussian intersection method to estimate the redox potential of thiol/disulfide pairs in 12 proteins belonging to the thioredoxin superfamily [44]. Our deviations from calculated values from experimental data are also in line with recently reported TI simulations carried out to estimate ligand binding affinity [32,45,46].

**Figure 3:**
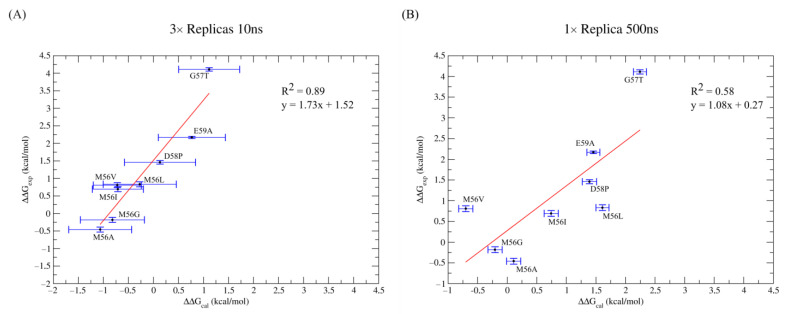
Scatter plots ΔΔG_cal_ Vs ΔΔG_exp_ for 3× replicas of 10ns (**A**) and for 1× replica of 500 ns (**B**). Trendlines are reported in red, which have a slope of 1.72 and 1.08 for 3 × 10 ns (**A**) and for 1 × 500 ns (**B**), respectively.

The 0.89 R^2^ value also indicates that the method satisfactorily estimates the differences in redox potential among the tested mutants (Figure 3). On the other hand, computed ΔΔG values obtained from a 500 ns trajectory are characterized by poorer agreement with experimental values; in fact, the R^2^ value is 0.58, although the minimum, maximum, and mean absolute error are equal to 0.07 kcal/mol, 1.96 kcal/mol, and 0.70 kcal/mol, respectively. These data highlight that the best predictions are obtained by reproducing multiple short-time replicas rather than a single longer simulation. While the average absolute error is smaller in the 500 ns simulation, the relative behavior of the data shows improvement in the case of three 10 ns replicas, as shown by the R^2^ value being closer to 1. This implies that, by applying a correction factor, the method is more effective in predicting the relative redox potential trends of different mutations when multiple short-time replicas are used.

The relative error calculated for ΔΔG appears to be smaller for the 500 ns simulation, but such an observation is biased by the different number of replicas carried out in the 500 and 10 ns protocols. In fact, if each 10 ns simulation is individually compared to the 500 ns simulation, it is observed that the error calculated by the BAR method is nearly identical in both setups (values are in a range from 0.04 to 0.08). The error increases when ΔΔG is calculated on the three concatenated 10 ns trajectories, suggesting that the BAR method is more affected by the number of independent trajectories than their duration.

**Figure 4:**
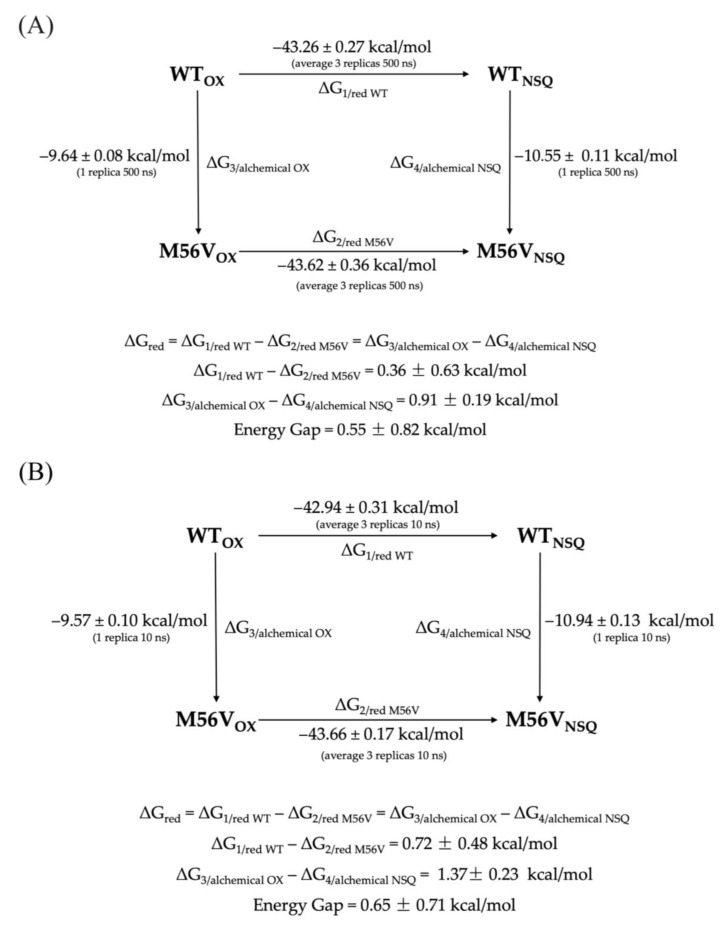
Thermodynamic cycle showing the free energy differences of reduction and the free energy differences of WT to M56V transformation for 3 × 500 ns (**A**) and 3 × 10 ns (**B**).

For selected systems, we have also produced replicas of 500 ns trajectories in order to analyze the consistency of the methodology, reproducibility, and influence of the number of fast transitions on the final ΔG value. In addition, to test the consistency of the results, we also evaluated whether the chosen thermodynamic cycle model (shown in Figure 4) closed properly. The chosen systems were the WT flavodoxin and the M56V mutant, as the latter is characterized by an unsigned error (UE) for the ΔΔG above 1 kcal/mol, i.e., the second highest calculated among all systems. The cycle was constructed using two protein mutagenesis reactions as vertical branches, where flavodoxin WT was chemically converted into its M56V variant, both in the oxidized form and in the semiquinone form of the flavin cofactor. The values of the horizontal branches (WT_OX_ → WT_NSQ_ and M56V_OX_ → M56V_NSQ_) were obtained with 3 MD runs of 500 ns each, while the vertical branches (WT_OX_ → M56V_OX_ and WT_NSQ_ → M56V_NSQ_) were obtained with a single 500 ns MD each. The same protocol is repeated using 10 ns MD simulations. Even if a statistical analysis on multiple mutants would be necessary to exclude the possibility that the discrepancy obtained is mutant-dependent, the results are very encouraging. In fact, the difference obtained between the horizontal and vertical branches is below 0.55 kcal/mol (using 500 ns MD simulations) and 0.65 kcal/mol (using 10 ns MD simulations), showing that the calculations are sound for this type of system and suggesting that the ΔΔG error associated with the redox reaction may mainly derive from an inadequate force field. Tests with other force fields will be carried out in the future to further test this hypothesis.

With the aim of providing a rationale for the difference in redox potential among wild-type and single-point mutant flavodoxins, we have also examined in detail the structural and dynamic properties of the flavin cofactor and of its environment. In particular, for such a purpose, four structural features were taken into account, both for OX and SQ states. (i) The distance between the centers of geometry (COGs) of the protein and the isoalloxazine ring; (ii) the distance between the COGs of the isoalloxazine ring and TRP90 that plays an important role in the affinity of FMN to the pocket [43]; (iii) the distance between N5 (HN5 in the case of SQ flavin) and carbonyl oxygen of residue 57; and (iv) the value of the ω-torsion angle of residues 57–58 (Figures S5 and S6; Supplementary Materials). When considering 3 × 10 ns trajectories, the investigated parameters do not undergo any significant change, possibly due to the short timescale (Figure S5; Supplementary Materials). Nevertheless, the calculated redox potentials well correlate with the experimental data. This observation suggests that the chemical properties of the environment surrounding the flavin cofactor have a significant impact on the redox potential, whereas structural rearrangement does not play an important role in redox potential modulation. When considering the 1 × 500 ns trajectory, the most relevant difference among the enzyme variants is the distance between the hydrogen H5 and the carbonyl oxygen of residue 57 (Ocarbonyl-Hflavin; Figure S6), which quantifies the extent of the formation of a hydrogen bond between the flavin moiety and residue 57. Single-point mutants G57T, E59A, and M56L are characterized by an increase in the Ocarbonyl-Hflavin distance along the MD simulations, and a corresponding decrease in the redox potential is computed, in nice agreement with previous proposals indicating that such a bond stabilizes the SQ state [13].

## 3. Materials and Methods

### 3.1. Simulated Systems

In this work, we explored the first reduction event in nine variants of *Clostridium Beijerinckii* flavodoxin (WT, G57T, D58P, E59A, M56A, M56G, M56L, M56I, and M56V). The chosen mutants are all electrochemically characterized, and the redox potentials associated with both flavin reductions are known [13,22,43]. The starting structures of WT, G57T, and D58P flavodoxins correspond to the X-ray oxidized (pdb id: 5NLL, 1FLD, 4NUL) and semiquinone (pdb id: 2FOX, 5NUL, 1FLN) structures. The crystallographic structure of WT_OX_ (5NLL) presents the three known alternative conformations for the 50’s loop [13]; only the structure prevailing in solution (cis-O-down) was selected for the simulations. The G57T_SQ_ crystal (5NUL) is also characterized by the presence of two conformers, of which only the prevalent one [13], featuring trans-O-up conformation, is used for the simulations. The pdb structure 1FLN corresponds to the fully reduced state of flavodoxin; however, due to the strong structural similarity between the SQ and HQ states found in the other crystallized structures, such a structure was also used for the semiquinone one. The starting structures of other systems are obtained by in silico mutagenesis of oxidized, semiquinone, and fully reduced X-ray structures of the WT system using the Pymol mutagenesis tool (Table 3).

All structures are processed with the protein preparation wizard of Maestro 2021-4 software to remove the co-crystallized water molecules, add hydrogen atoms assuming pH = 7, and fill in missing side chain atoms and missing loops.

### 3.2. MD Simulations

All equilibrium molecular dynamics simulations were produced using the GROMACS 2020.3 [47] software and the CHARMM36 [48] force field integrated with the parameters for flavins developed by Alexey Aleksandrov [49]. The investigated flavoproteins were solvated with TIP3P water molecules [50] in a cubic box of 6.22 nm^3^. Counter ions K^+^ and Cl^-^ were added to obtain a neutrally charged box and to reach a salt concentration of 0.1 M. All models were minimized using a steepest descent algorithm and then equilibrated at 300 K and 1 bar through a 4 ns NVT simulation and 1ns NPT simulation, respectively. Coupling to the external bath is achieved using two different algorithms: the modified Berendsen thermostat is used for temperature [51,52] and the Parrinello–Rahman algorithm for pressure [53,54]. The LINCS algorithm [55] was used to constrain all covalent bonds in which hydrogen atoms are involved, while the equations of motion are integrated using the leap-frog algorithm. The non-covalent interactions were computed according to the Verlet cutoff scheme [56], while van der Waals interactions and short-range electrostatic interactions were evaluated at each time step within 1.2 nm of each particle. Long-range electrostatic interactions were evaluated using the PME method with an interpolation order of 4 and the remaining parameters set to default values [57]. All simulations were performed using periodic boundary conditions in all directions. The production simulations were carried out using an integration time step of 1 fs. We initially tested a first protocol of three MD simulations of 10 ns followed by three TI simulations (similarly to what has been performed by Aldeghi et al. [32]); then, we tried another approach in which a single MD simulation of 500 ns was followed by one TI calculation in order to evaluate the effect of extended sampling on the free energy estimation.

### 3.3. TI Simulations

Hybrid topologies were generated using the PMX package developed by Daniel Seelinger and Bert L. de Groot [58]. A total of 500 equidistant snapshots were extracted from each MD simulation for each system (in the case of 3 × 10 ns simulations, 500 snapshots were extracted from each replica), and they were used as a starting point for 100 ps non-equilibrium transitions. These simulations were carried out using a Δλ of 10^−5^ and setting the sc-alpha and sc-sigma to 1.2 and 0.3 [44], respectively. Finally, the ΔG corresponding to the redox reactions was calculated using the Bennet Acceptance Ratio (BAR) approach [59]. When considering the 3 × 10 ns simulations, the BAR method was also applied to the three forward and three reverse concatenated trajectories. Uncertainties were estimated as standard errors (σ_ΔG_) by separately considering each equilibrium simulation and its related non-equilibrium trajectories as independent calculations (i.e., when 3 equilibrium simulations were used, 3 independent ΔG values were obtained, and these were used to estimate σ_ΔG_). These uncertainties were then propagated to the final ΔΔ𝐺 estimate so as to obtain the standard error σ_ΔΔG_. The total amount of time simulated in this work is about 21 μs.

## 4. Conclusions

In this study, we have shown that fast growth-TI calculations can reliably be used to predict even subtle thermodynamic properties, such as the difference in redox potentials for WT and single-point mutant flavodoxins. Therefore, fast growth-TI calculations have the potential to be a robust computational tool available to research laboratories for the design of flavoproteins with tailored redox properties. It remains to be evaluated how this computational approach remains effective for other redox enzyme families and/or proton-coupled redox reactions where not only the cofactor but also proximal aminoacids are directly involved in the process. Studies aimed at investigating such issues are currently underway in our laboratory.

## Figures and Tables

**Table 1:** Free energy values of the first reduction in wild-type flavodoxin considering all the 50’s loop conformation for the OX.

WT	Exp. Proportion	Replica ^a^	${∆G}_{cal}\;10\;ns$ ^b^	$\mathrm{Average}\;{∆G}_{cal}$ ^b,c^	Standard Error ^b^	MAE ^b^	RMSE ^b^
Cis-O-down	50%	1	−43.31 ± 0.05	−42.92	0.31	1.37	1.54
		2	−43.44 ± 0.04				
		3	−42.48 ± 0.04				
Trans-O-down	20%	1	−42.92 ± 0.05	−42.91	0.19	0.64	0.87
		2	−43.97 ± 0.04				
		3	−43.73 ± 0.05				
Trans-O-up	30%	1	−43.17 ± 0.05	−43.76	0.32	1.37	1.54
		2	−42.70 ± 0.04				
		3	−43.33 ± 0.04				
			Overall	−43.17 ^d^	0.29 ^d^	1.06 ^e^	1.25 ^e^

(^a^) 10 ns simulations. (^b^) Values are reported in kcal/mol. (^c^) ΔG is calculated using the BAR method on the three concatenated trajectories. (^d^) All values are calculated as the weighted arithmetic mean using the experimental relative abundance as weight. (^e^) MAE and RMSE are calculated using the overall ΔG as reference.

**Table 2:** ΔG and ΔΔG one-electron reduction potentials estimate both experimentally (exp) and in silico (calc) for 3 × 10 ns and 1 × 500 ns simulations.

3 × 10 ns Simulations								
	${∆G}_{exp}$ ^a^	${∆∆G}_{exp}$ ^a,b^	${Replica1}_{calc}$ ^a^	${Replica2}_{calc}$ ^a^	${Replica3}_{calc}$ ^a^	${∆G}_{calc}$ ^a,c,d^	${∆∆G}_{calc}$ ^a,b^	Absolute Error ^a^
WT	2.12 ± 0.11	-	−43.33 ± 0.05	−43.46 ± 0.04	−42.50 ± 0.04	−42.94 ± 0.31	-	-
G57T	6.23 ± 0.11	4.11 ± 0.23	−41.17 ± 0.04	−42.12 ± 0.05	−42.05 ± 0.04	−41.83 ± 0.30	1.11 ± 0.61	3.00
D58P	3.58 ± 0.11	1.46 ± 0.23	−41.98 ± 0.05	−42.81 ± 0.06	−43.31 ± 0.03	−42.81 ± 0.40	0.13 ± 0.71	1.33
E59A	4.29± 0.12	2.17 ± 0.23	−41.64 ± 0.04	−42.53 ± 0.05	−43.22 ± 0.05	−42.18 ± 0.36	0.76 ± 0.67	1.41
M56A	1.66 ± 0.18	−0.46 ± 0.30	−43.98 ± 0.04	−43.66 ± 0.06	−44.56 ± 0.05	−44.01 ± 0.32	−1.06 ± 0.63	0.60
M56G	1.94 ± 0.18	−0.18 ± 0.30	−44.14 ± 0.06	−43.31 ± 0.05	−44.45 ± 0.07	−43.76 ± 0.33	−0.82 ± 0.64	1.24
M56L	2.95 ± 0.18	0.83 ± 0.30	−42.93 ± 0.06	−43.14 ± 0.05	−44.20 ± 0.05	−43.21 ± 0.42	−0.27 ± 0.73	1.10
M56I	2.81± 0.18	0.69 ± 0.30	−43.18 ± 0.07	−43.93 ± 0.06	−43.68 ± 0.08	−43.65 ± 0.20	−0.71 ± 0.51	1.40
M56V	2.93 ± 0.18	0.81 ± 0.30	−43.95 ± 0.05	−43.61 ± 0.05	−43.49 ± 0.05	−43.66 ± 0.17	−0.72 ± 0.48	1.53
						MAE	1.37	
						RMSσ	0.06	
						RMSE	1.54	
1 × 500 ns Simulations								
	${∆G}_{exp}$ ^a^		${∆∆G}_{exp}$ ^a,b^	${∆G}_{calc}$ ^a^		${∆∆G}_{calc}$ ^a,b^		Absolute Error ^a^
WT	2.12 ± 0.11		-	−43.60 ± 0.08		-		-
G57T	6.23 ± 0.11		4.11 ± 0.23	−41.45 ± 0.04		2.15 ± 0.12		1.96
D58P	3.58 ± 0.11		1.46 ± 0.23	−42.21 ± 0.04		1.39 ± 0.12		0.07
E59A	4.29± 0.12		2.17 ± 0.23	−42.14 ± 0.04		1.46 ± 0.12		0.71
M56A	1.66 ± 0.18		−0.46 ± 0.30	−43.49 ± 0.04		0.11 ± 0.12		0.57
M56G	1.94 ± 0.18		−0.18 ± 0.30	−43.80 ± 0.05		−0.20 ± 0.13		0.02
M56L	2.95 ± 0.18		0.83 ± 0.30	−41.99 ± 0.03		1.61 ± 0.11		0.78
M56I	2.81± 0.18		0.69 ± 0.30	−42.86 ± 0.05		0.74 ± 0.13		0.05
M56V	2.93 ± 0.18		0.81 ± 0.30	−44.30 ± 0.04		−0.70 ± 0.12		1.51
				MAE		0.70		
				RMS		0.95		

(^a^) Values are reported in kcal/mol. (^b^) ΔΔG are calculated respect to WT. (^c^) Average of Replica1, Replica2 and Replica3. (^d^) ΔG is calculated using the BAR method on the three concatenated trajectories.

**Table 3:** Summary of simulation system details of the OX and NSQ flavin-flavodoxin complex.

Sistem	OX		NSQ	
	Structure ^a^	50’s Loop ^b^	Structure ^a^	50’s loop ^b^
WT	5NLL	cis-O-down	2FOX	trans-O-up
G57T	1FLD	trans-O-down	5NUL	trans-O-down
D58P	4NUL	cis-O-down	5FLN	trans-O-down
E59A	5NLL in silico mutagenesis	cis-O-down	2FOX in silico mutagenesis	trans-O-up
M56A	5NLL in silico mutagenesis	cis-O-down	2FOX in silico mutagenesis	trans-O-up
M56G	5NLL in silico mutagenesis	cis-O-down	2FOX in silico mutagenesis	trans-O-up
M56L	5NLL in silico mutagenesis	cis-O-down	2FOX in silico mutagenesis	trans-O-up
M56I	5NLL in silico mutagenesis	cis-O-down	2FOX in silico mutagenesis	trans-O-up
M56V	5NLL in silico mutagenesis	cis-O-down	2FOX in silico mutagenesis	trans-O-up

(^a^) PDB code. (^b^) Conformation of the reverse turn involving residues 56–59 in the Clostridium Beijerinckii flavodoxin.

## Data Availability

Not applicable.

## Supplementary Materials

The following supporting information can be downloaded at: https://www.mdpi.com/article/10.3390/molecules28166016/s1.
