# Supplementary material for: Assessing the Performance of Non-Equilibrium Thermodynamic Integration in Flavodoxin Redox Potential Estimation
Source: Molecules. 2023 Aug 11;28(16):6016. doi: 10.3390/molecules28166016 (PMC10459689; doi:10.3390/molecules28166016)

Figure S1. RMSD values of Wild-type and mutant variants for 3x10ns replicas (A) and 1x500ns replica (B). Red vertical lines in panel A divide the total RMSD showing the three replicas for each flavodoxin.

A)

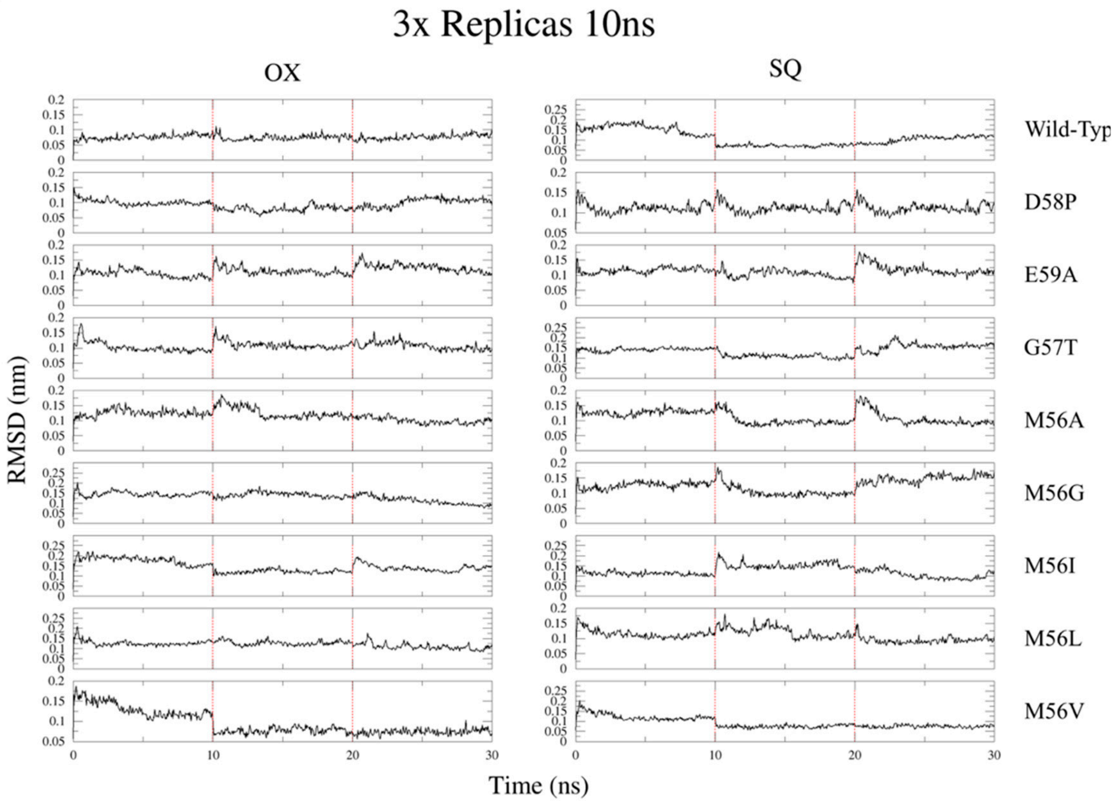

B)

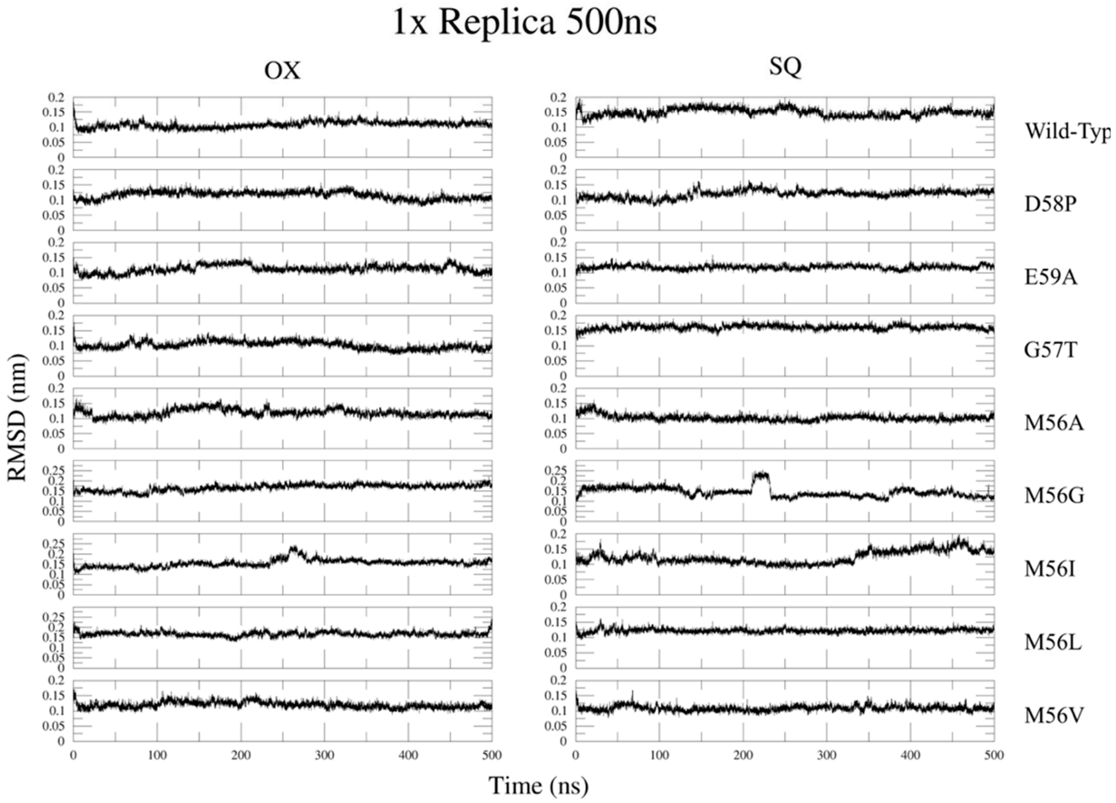

Figure S2. RMSF values of Wild-Type and mutant variants for 3x10ns replicas (A) and 1x500ns replica (B)

A)

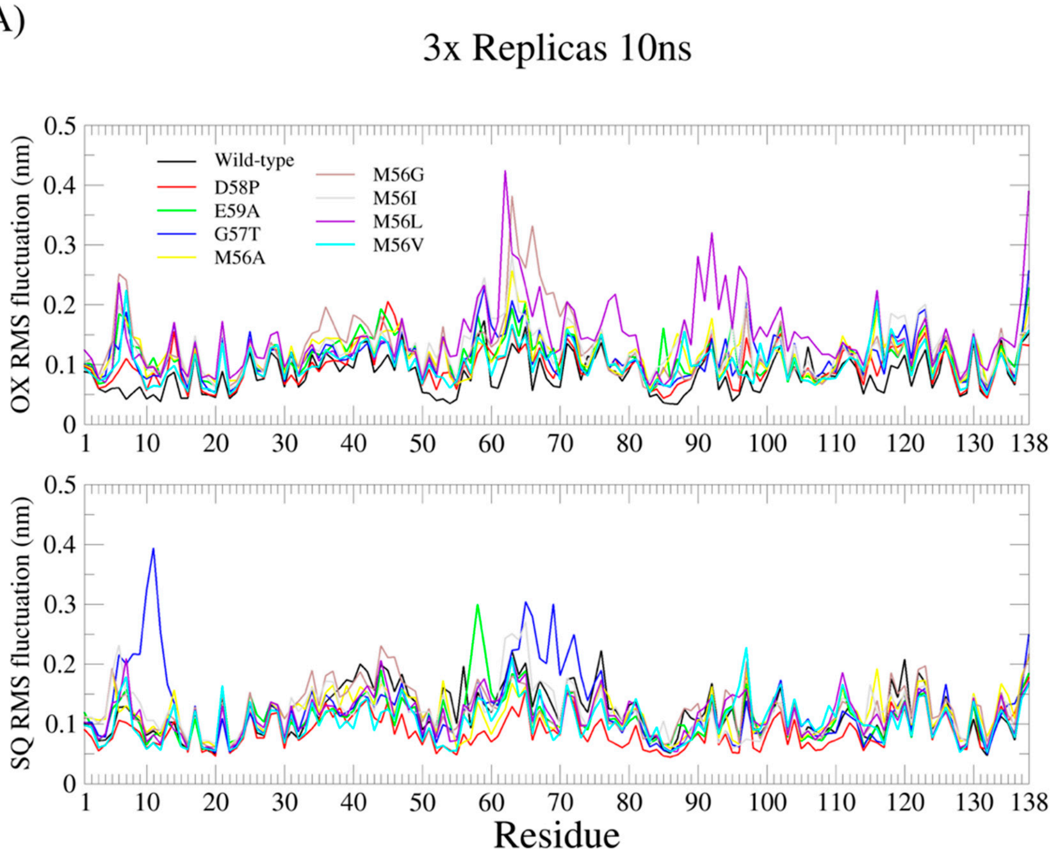

B)

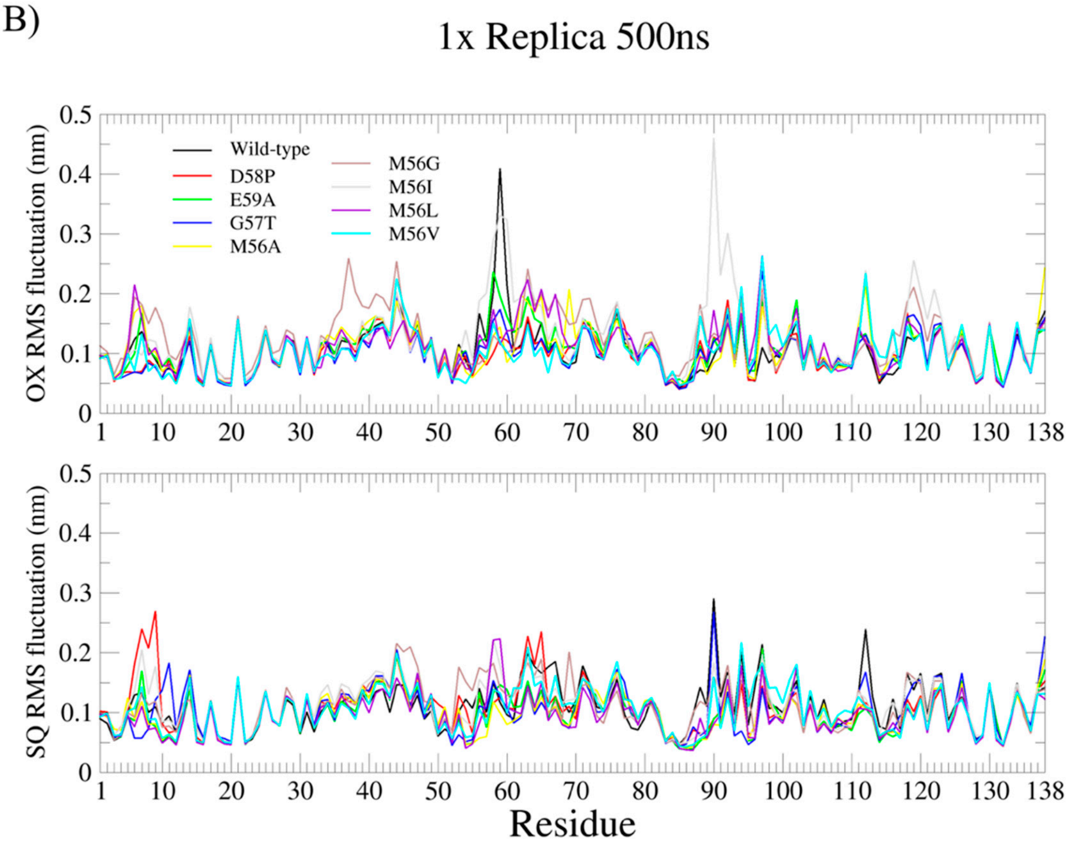

Figure S3. ΔG values calculated with the BAR estimator for the nine flavodoxin variants in the 3x10ns simulations. For each system, ΔG value is calculated taking together all the transitions collected in the three replicas; as a result 1500 transitions are used for both forward and backward reaction. On the left, the work values for the forward (green) and backward (blue) transitions are reported for every starting structure. On the right, the distributions of these work values are shown as histograms and are used to draw the gaussians (green and blue dashed lines), the intersection (black dashed line) of which allows obtaining the free energy difference.

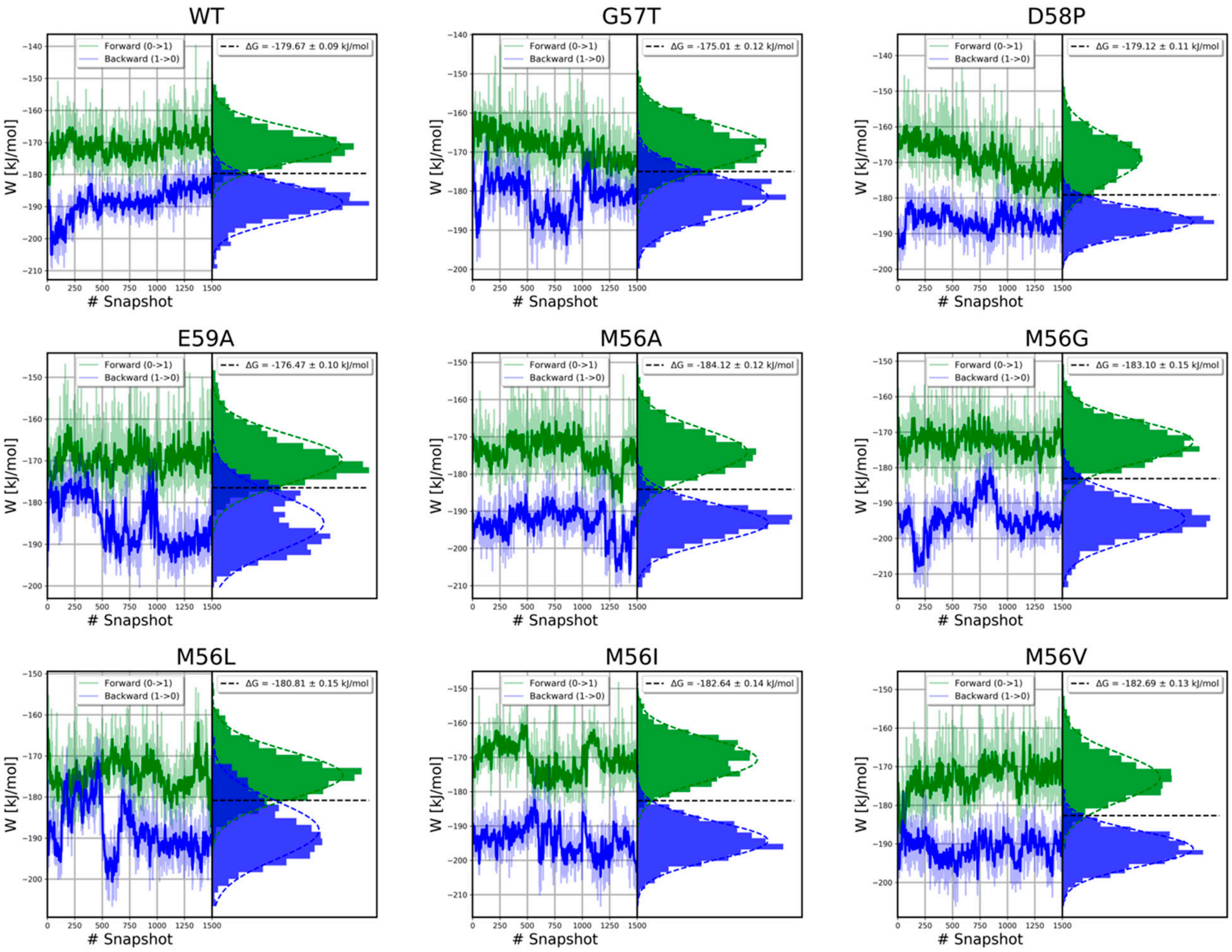

Figure S4. ΔG values calculated with the BAR estimator for the nine flavodoxin variants in the 1x500ns simulations. For each system ΔG value is calculated using 500 transitions for both forward and backward reaction. On the left, the work values for the forward (green) and backward (blue) transitions are reported for every starting structure. On the right, the distributions of these work values are shown as histograms and are used to draw the gaussians (green and blue dashed lines), the intersection (black dashed line) of which allows obtaining the free energy difference.

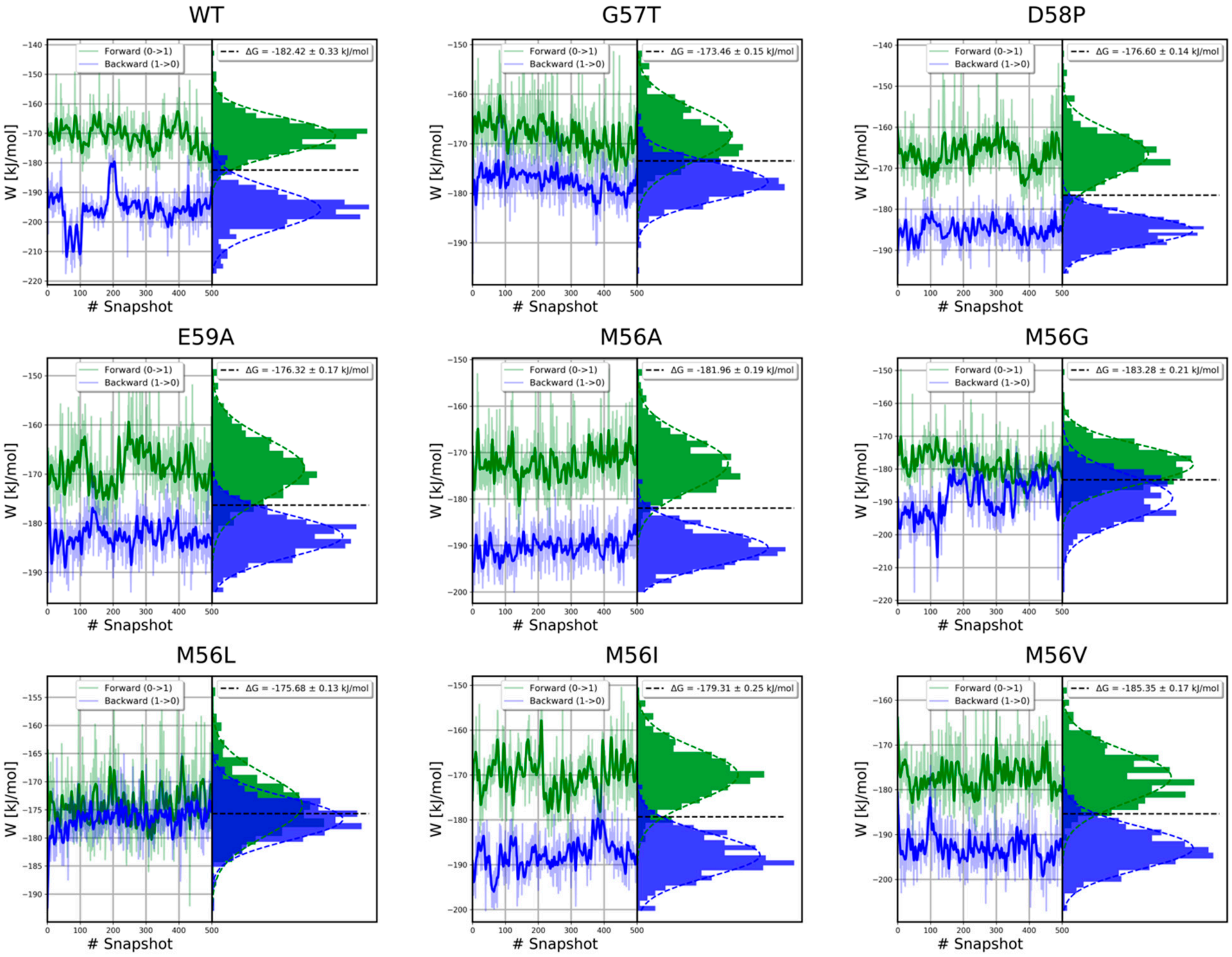

Figure S5. Structural data for wild-type and mutant flavodoxins in the 3x10ns simulations.

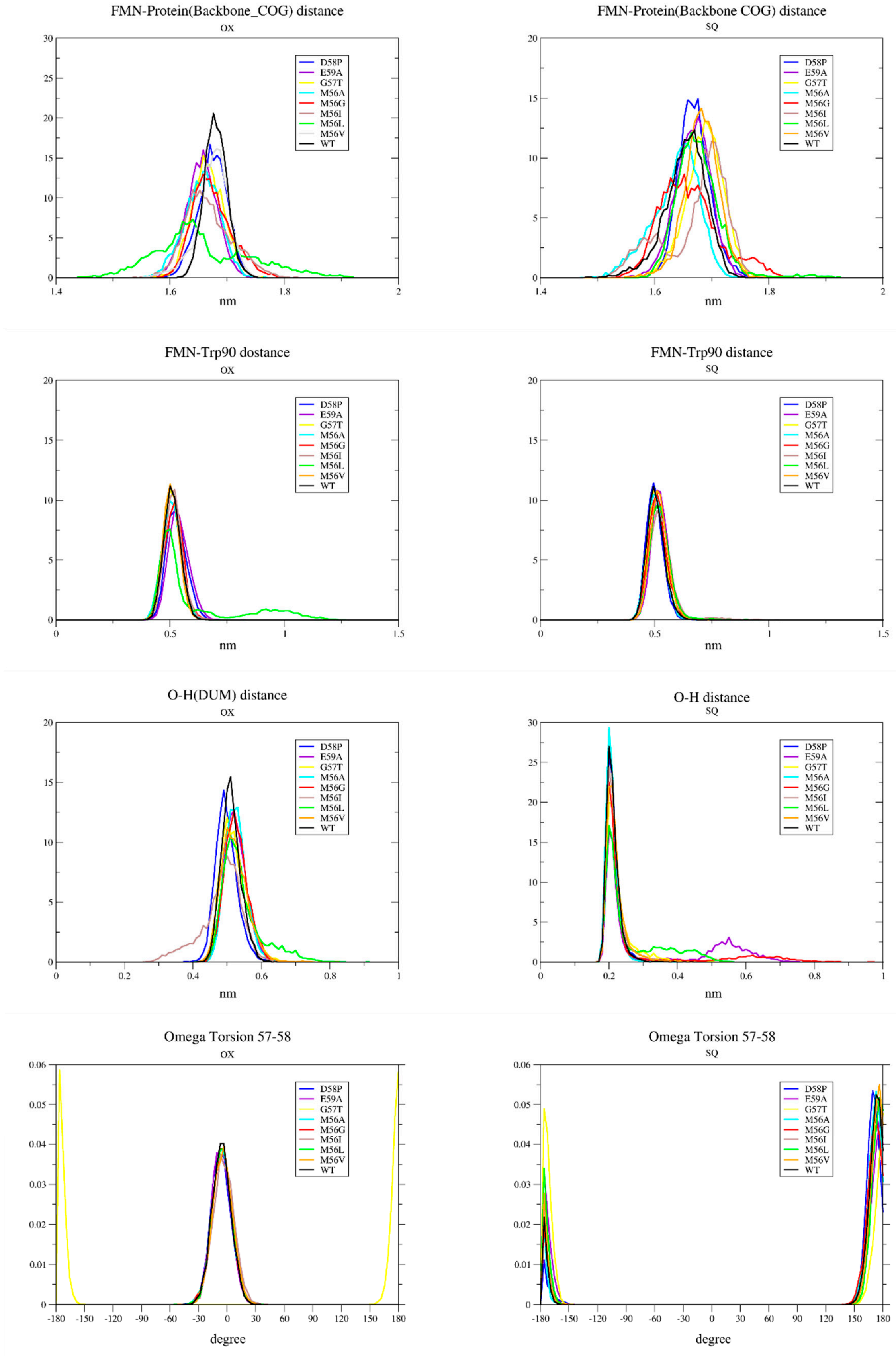

Figure S6. Structural data for wild-type and mutant flavodoxins in the 1x500ns simulations.

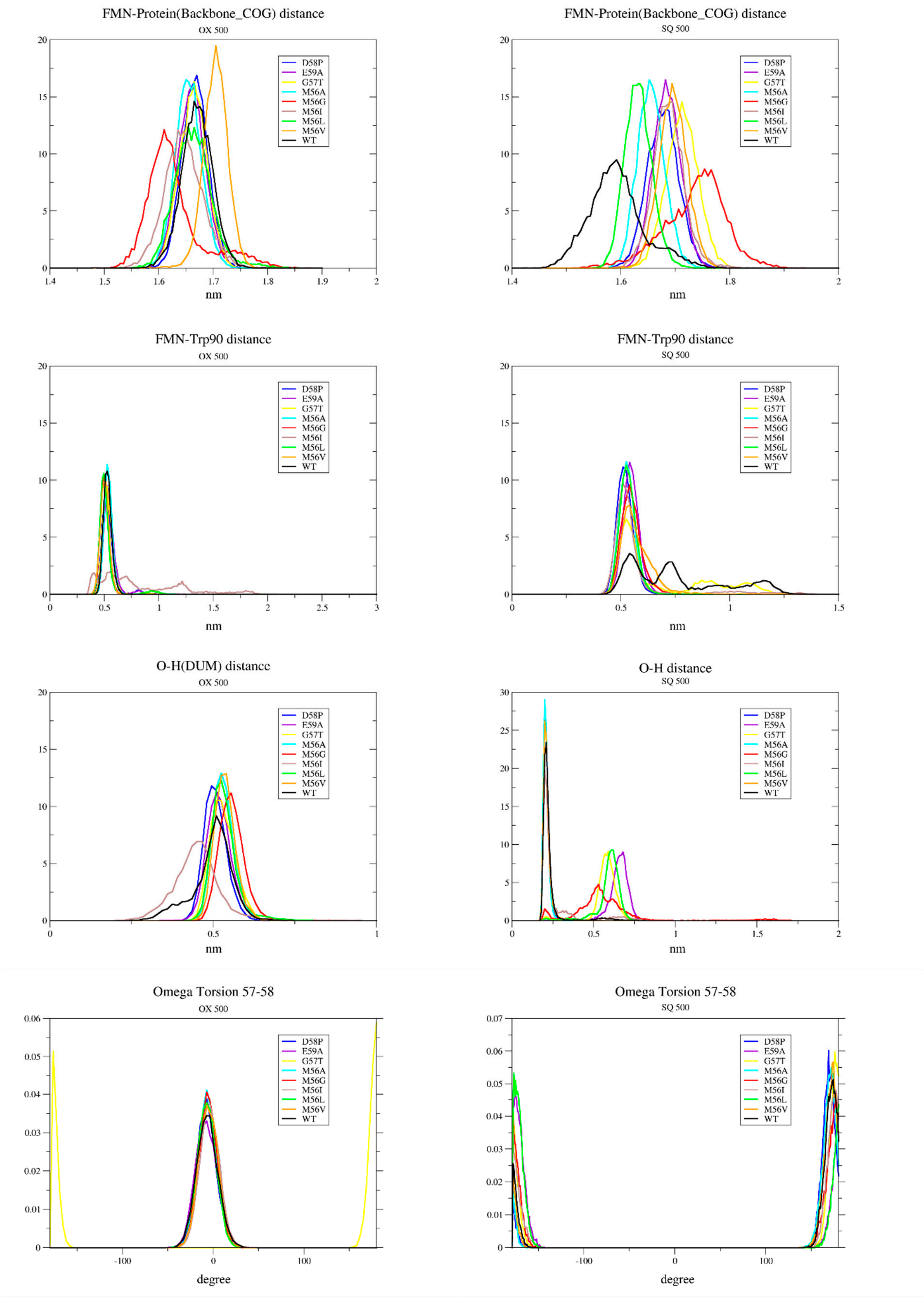

Supplement: Supplementary file 1 [file molecules-28-06016-s001.zip › molecules-2507850-supplementary.pdf]
